# New Advances in Urea Transporter UT-A1 Membrane Trafficking

**DOI:** 10.3390/ijms140510674

**Published:** 2013-05-21

**Authors:** Guangping Chen

**Affiliations:** Department of Physiology, Emory University School of Medicine, Atlanta, GA 30322, USA; E-Mail: gchen3@emory.edu; Tel.: +1-404-727-7494; Fax: +1-404-727-2648.

**Keywords:** lipid rafts, glycosylation, accessory proteins, SNARE protein, cytoskeleton protein

## Abstract

The vasopressin-regulated urea transporter UT-A1, expressed in kidney inner medullary collecting duct (IMCD) epithelial cells, plays a critical role in the urinary concentrating mechanisms. As a membrane protein, the function of UT-A1 transport activity relies on its presence in the plasma membrane. Therefore, UT-A1 successfully trafficking to the apical membrane of the polarized epithelial cells is crucial for the regulation of urea transport. This review summarizes the research progress of UT-A1 regulation over the past few years, specifically on the regulation of UT-A1 membrane trafficking by lipid rafts, *N*-linked glycosylation and a group of accessory proteins.

## 1. Introduction

Urea is the major end product of amino acid metabolism. It is generated from the ornithine cycle in liver, and is ultimately excreted by the kidney representing 90% of total nitrogen in urine. The physiological significance of urea in the production of concentrated urine was recognized by Gamble in the 1930s [1,2]. Urea reabsorbed in the kidney inner medullary collecting duct (IMCD) contributes to the development of the osmolality in the medullary interstitium. The osmotic gradient, along the corticomedullary axis in the kidney, allows the water and important solutes to be reabsorbed from the renal tubules back into the interstitium and, thereby, generates concentrated urine.

Urea is a small but highly polar molecule with a low permeability across cell membranes [1]. Urea transport across the cell membrane is mediated by the specialized urea transporter (UT). The first UT cDNA was cloned in 1993 [3]. To date, two mammalian UT subfamilies have been reported, including the renal tubular type urea transporter UT-A and the erythrocyte vascular-type urea transporter UT-B [1,4]. In kidneys, four types of UT-A isoforms have been identified. UT-A1 and UT-A3 are found in the inner medullary collecting duct; UT-A2 is located in the thin descending limb, and UT-A4 is only detected at the mRNA level in rat kidneys. UT-A1 is the largest protein isoform and is expressed in the apical plasma membrane of the IMCD [5]. Physiological studies in UT-A1/UT-A3 knockout mice demonstrate the essential role of these urea transporters in the urinary concentrating mechanism [6].

The regulation of UT-A1 is complicated and occurs at different levels, including gene transcription, protein expression, and posttranslational modification. Arginine vasopressin (also known as antidiuretic hormone) is the major hormone that regulates UT-A1 transport activity *in vivo* [7,8]. In addition, UT-A1 has also been shown to be regulated by angiotensin II and oxytocin [9,10]. Vasopressin increases kidney urea transport activity by increasing UT-A1 in the apical plasma membrane [10] and UT-A1 phosphorylation [8,11]. Like all transporters at the plasma membrane, UT-A1 function is highly dependent on its membrane trafficking and recent discoveries indicate that lipid rafts, *N*-linked glycosylation and interacting proteins (such as caveolin, snapin and actin) play key roles in this process.

## 2. Role of Lipid Rafts

**Figure 1:**
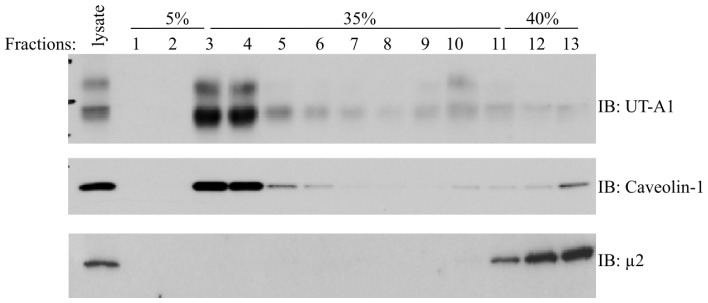
Cell surface UT-A1 is concentrated in lipid raft microdomains. Rat kidney inner medullary collecting duct (IMCD) suspension was lysed in 1% Triton X-100 and loaded on 5%–40% sucrose gradient for lipid raft isolation. After 20 h centrifugation, equal amount of fractions were collected from the top to bottom and Western blotted for UT-A1. Caveolin-1 was used as a positive control for the lipid raft fraction. Clathrin mu2 was used as an indicator for non-lipid raft fractions. Reproduced from Feng *et al*. (2009) [14].

The plasma membrane contains many specialized microdomains, referred to as lipid rafts. The lipid raft is a highly ordered but transient membrane structure enriched in cholesterol and sphingolipids [12]. Numerous proteins are associated with and regulated by association with lipid rafts in the cell membrane. Lipid rafts have been implicated in the regulation of intracellular protein trafficking [13]. By using non-ionic detergents Triton X-100 or Brij96 and sucrose gradient ultracentrifugation, it was found that the cell membrane UT-A1 is concentrated in the lipid raft microdomains and co-distributed with caveolin-1 from rat kidney IMCD (Figure 1) [14,15] as well as UT-A1 transfected HEK293 and MDCK cells [15–17]. In polarized epithelial cells, lipid rafts are found primarily in the apical membrane. Therefore, targeting to lipid raft microdomains is important for membrane protein apical trafficking in polarized epithelial cells [18,19]. It has been shown that differential partitioning into lipid raft microdomains is an important determinant of the apical localization of the plasma membrane calcium ATPase (PMCA) 2w and 2z splice variants [20]. UT-A1 association with lipid rafts also represents an important regulatory mechanism for UT-A1 apical membrane targeting.

## 3. Role of *N*-Glycosylation

**Figure 2:**
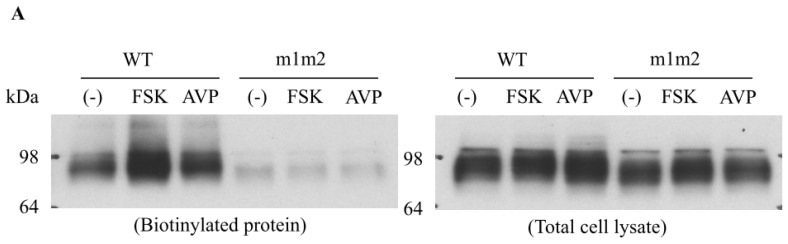

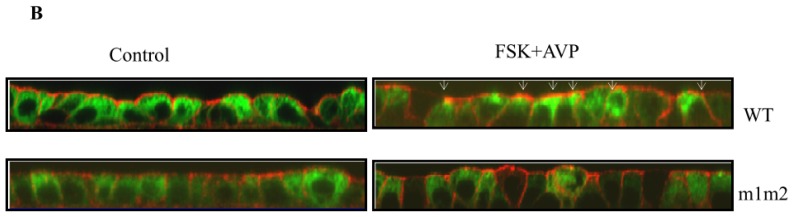
*N*-Glycosylation is required for UT-A1 membrane trafficking. (**A**) Cell surface biotin labeling. Cells were grown in a six-well plate to confluence and then treated with either 10 nM AVP, 10 μM FSK or without any treatment for 15 min. Cell surface proteins were biotinylated, recovered with streptavidin-beads and analyzed by Western blot. The left panel shows membrane proteins collected on streptavidin beads. The right panel shows pre-bead samples (total proteins); (**B**) Immunofluorescence microscopy. Cells were grown on a Transwell insert filter for three days to allow the cells to develop polarity and then treated with 10 nM AVP and 10 μM FSK for 15 min. The cells were processed for immunostaining with UT-A1 antibody and fluorescein isothiocyanate-conjugated secondary goat anti-rabbit antibody. F-actin was visualized by Texas Red-X Phalloidin (Molecular Probes). Reproduced from Chen *et al.* (2006) [26].

Most membrane proteins targeted to the plasma membrane possess *N*-linked glycans [21–23]. Glycosylation serves as an important apical membrane trafficking signal that has been well acknowledged [24,25]. It has been shown that membrane proteins, such as AQP2 [23], NCC [21], are unable to exit the Golgi apparatus and failed to be delivered to the cell membrane when they are unglycosylated. Urea transporter UT-A1 is a heavily glycosylated protein with two glycosylation sites at Asn279 and Asn742 [26]. Immunoblotting studies of UT-A1 in normal rat renal inner medulla reveal a predominant protein band of 97 kDa and a less abundant protein band of 117 kDa; both bands represent glycosylated versions of the 88-kDa UT-A1 protein core [27]. The glycosylation state of UT-A1 is directly linked to urea transporter activity. A tubule perfusion study by Pech *et al.*, shows that the appearance of the 117-kDa form in the inner medullary base is associated with increased urea transport activity [28]. Using site-specific mutagenesis to remove *N*-glycan consensus sites, it was found that *N*-glycosylation is critical for UT-A1 membrane trafficking and stability [26]. UT-A1 lacking the two glycosylation sites has significantly impaired cell membrane expression (Figure 2) and reduced transport activity [26]. Loss of glycosylation affects UT-A1 exiting both the ER and Golgi, particularly affecting its movement from the Golgi complex to the cell surface [26].

**Figure 3:**
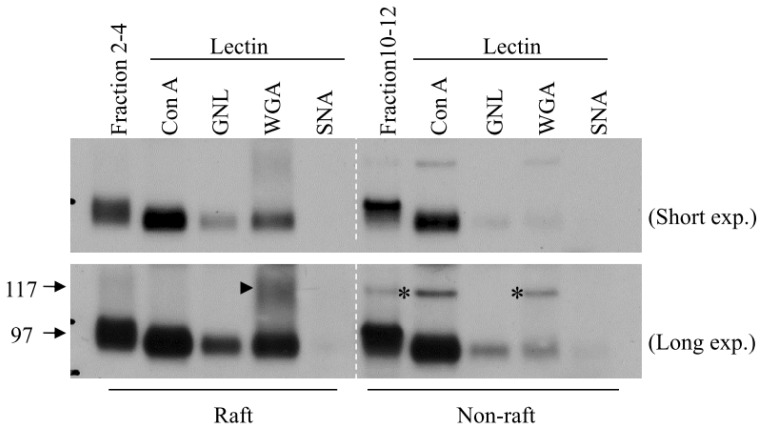
Mature glycosylated UT-A1 targets in lipid rafts. Lectin pulldown assays with UT-A1 MDCK cell samples. Lipid raft (fractions 2–4) and nonraft fractions (fractions 10–12) of UT-A1 MDCK cells were incubated with the indicated agarose-bound lectins at 4 °C overnight. After washing, the precipitated samples were analyzed by Western blotting with UT-A1 antibody. Bottom panel with deliberately extended exposure time shows the 117-kDa glycosylation band (arrowhead). Con A: concanavalin A; GNL: Galanthus nivalis lectin; WGA: wheat germ agglutinin; SNA: Sambucus nigra lectin. Asterisk indicates nonspecific band. Reproduced from Chen *et al.* (2011) [15].

Although the role of *N*-linked glycosylation in membrane protein trafficking has been recognized for many years, the underlying mechanism remains unclear. New findings show that the maturation of *N*-linked glycosylation is associated with the UT-A1 trafficking into membrane lipid raft subdomains [15]. The mature *N*-linked glycosylation, particularly with high amounts of *N*-acetylglucosamine and sialic acid, facilitates UT-A1 lipid raft membrane localization. In contrast, immature glycosylated UT-A1 with a high amount of mannose is in the non-lipid raft fractions (Figure 3) [15]. These findings suggest that future study should investigate the role of the glycan structure which could provide new information revealing how glycosylation regulates membrane protein trafficking. In terms of this, the new technology of glycomics will be helpful to decipher the specific oligosaccharide structures involved in the regulation of UT-A1 apical membrane targeting and lipid raft association.

## 4. Role of Accessory Proteins

Membrane proteins are produced in endoplasmic reticulum, remodeled in the Golgi complex, and then targeted to the cell surface. In the journey of the membrane protein to the cell surface, many protein interactions are involved. These proteins act in concert to assure the specificity of the membrane protein in sorting, membrane trafficking, retrieval and functional activity. A number of the UT-A1 associated proteins have been found to assist UT-A1 membrane trafficking.

### 4.1. Caveolin

Many proteins found in lipid raft microdomains are associated with caveolin [29,30]. There are three caveolin isoforms. Caveolin-1 is the most ubiquitously expressed. It is found in the kidney, lung, heart, and brain. Caveolin-2 facilitates but is not essential for caveolae formation. Caveolin-3 is specific to muscle [29,31]. Besides serving as the structural protein core of caveolae, caveolin protein acts as a scaffold and plays an important role in recruiting numerous proteins into lipid rafts [31,32]. Urea transporter UT-A1 also interacts with caveolin-1 in lipid rafts [14], which provides another mechanism for the regulation of UT-A1 trafficking to lipid raft domains within the plasma membrane.

### 4.2. Snapin

Snapin is a ubiquitously expressed SNARE (soluble *N*-ethylmaleimide sensitive fusion protein attachment protein receptor)-associated protein. SNAREs are a superfamily of small, mostly membrane associated proteins, essential for all intracellular membrane fusion steps [33]. SNAREs associated with the plasma membrane are involved in both regulated and constitutive exocytosis [33]. Snapin is directly associated with native UT-A1 from kidney IMCD [34]. The *C*-terminal coiled-coil domain (H2) of snapin mediates its interaction with the large intracellular loop of UT-A1. Meanwhile, snapin also binds syntaxin 4 and SNAP23, the two major components of target-SNARE machinery [34]. Snapin mediates UT-A1 connection with the SNARE machinery, suggesting that the SNARE mediated vesicle trafficking mechanism may be functionally important for regulating urea transport. Indeed, facilitation of UT-A1 coupling with SNARE proteins (snapin, syntaxin 4, and SNAP23) has been shown to increase UT-A1 membrane expression and urea transport activity [34].

Interestingly, SNARE complexes are also enriched in lipid raft microdomains and their lipid raft association is mediated by the palmitoylated SNAP-23 [13,35,36]. Raft membranes, but not non-raft membranes, are the sites of SNARE dependent membrane fusion [13]. In RBL mast cells, stimulation specifically increases the amount of SNARE complexes in lipid raft microdomains and supports the hypothesis that lipid rafts serve as sites of secretory granule exocytosis in RBL mast cells. It will be important to examine whether snapin association promotes UT-A1 lipid raft trafficking in the future.

### 4.3. Actin

The actin cytoskeleton is the basic cell’s structural component and is vital to the function of all eukaryotic cells. Not only does actin act as a structural protein or a simple scaffold protein, actin is also required for mitosis, cytokinesis, cell motility, muscle contract, maintenance of cell shape, endocytosis, and secretion. In polarized epithelial cells, the cortical filamentous actin beneath the apical membrane has been implicated in the regulation of transporter protein membrane expression [37]. Urea transporter UT-A1 directly associates with actin [17], as reported for many other transporter proteins such as the water channel AQP2 [38], the epithelial sodium channel ENaC [39], the cystic fibrosis transmembrane conductance regulator CFTR [40], the Na-K-2Cl cotransporter NKCC1 [41], cardiac l-type Ca channels [42], and the glucose transporter GLUT4 [43]. Presumably, actin, together with actin-associated proteins, may cross-link forming networks and function as a barrier for transporter protein trafficking between the apical membrane and the intracellular compartments. In terms of this, depolymerization of cortical F-actin is an important prerequisite for protein membrane trafficking. Xu *et al*. found that depolymerization of actin by latrunculin facilitates forskolin stimulated UT-A1 trafficking to the cell surface [17]. There is also increased AQP2 trafficking to the cell membrane when filamentous actin is disrupted [44]. Therefore, in response to stimulation, actin could modulate its polymerization state and regulate UT-A1 membrane trafficking. Interestingly, some actin has been identified in lipid rafts [17], consistent with the possibility that actin may also be involved in UT-A1 lipid raft trafficking.

## 5. Conclusion

The vasopressin-mediated UT-A1 trafficking to the plasma membrane is crucial for the regulation of UT-A1 activity. Although it may share some common mechanisms of protein membrane trafficking, recent studies reveal that UT-A1 trafficking may have its own unique regulation. In the cell membrane, UT-A1 is specifically targeted to lipid raft microdomains. Therefore, regulation of UT-A1 delivery to the lipid rafts is of particular importance for UT-A1 trafficking to polarized epithelial cells. Evidence has shown that mature *N*-linked glycosylation with *N*-acetylglucosamine and sialic acid facilitates UT-A1 targeting to cell membrane lipid rafts. Furthermore, a group of UT-A1 accessory proteins such as caveolin, snapin and actin, either residing in or relating to lipid rafts, modulate UT-A1 trafficking to cell membrane lipid raft domains. It will also be interesting to know the membrane trafficking mechanism for other urea transporters (UT-A2, UT-A3 and UT-B). Similar to the UT-A1, UT-A3 is highly expressed in lipid rafts in the cell membrane [45] and phosphorylation increases UT-A3 membrane accumulation [46]. However, so far little is known about UT-A2 and UT-B trafficking. Further studies are required to address this issue.

## References

[1] Sands J.M. (2007). Critical role of urea in the urine-concentrating mechanism. J. Am. Soc. Nephrol.

[2] Gamble J.L., McKhann C.F., Butler A.M., Tuthill E. (1934). An economy of water in renal function referable to urea. Am. J. Physiol.

[3] You G., Smith C.P., Kanai Y., Lee W.-S., Stelzner M., Hediger M.A. (1993). Cloning and characterization of the vasopressin regulated urea transporter. Nature.

[4] Klein J.D., Blount M.A., Sands J.M. (2012). Molecular mechanisms of urea transport in health and disease. Pflugers. Arch.

[5] Nielsen S., Terris J., Smith C.P., Hediger M.A., Ecelbarger C.A., Knepper M.A. (1996). Cellular and subcellular localization of the vasopressin-regulated urea transporter in rat kidney. Proc. Natl. Acad. Sci. USA.

[6] Fenton R.A., Chou C.L., Stewart G.S., Smith C.P., Knepper M.A. (2004). Urinary concentrating defect in mice with selective deletion of phloretin-sensitive urea transporters in the renal collecting duct. Proc. Natl. Acad. Sci. USA.

[7] Wall S.M., Han J.S., Chou C.L., Knepper M.A. (1992). Kinetics of urea and water permeability activation by vasopressin in rat terminal IMCD. Am. J. Physiol.

[8] Zhang C., Sands J.M., Klein J.D. (2002). Vasopressin rapidly increases phosphorylation of UT-A1 urea transporter in rat IMCDs through PKA. Am. J. Physiol. Renal Physiol.

[9] Kato A., Klein J.D., Zhang C., Sands J.M. (2000). Angiotensin II increases vasopressin-stimulated facilitated urea permeability in rat terminal IMCDs. Am. J. Physiol. Renal Physiol.

[10] Klein J.D., Fröhlich O., Blount M.A., Martin C.F., Smith T.D., Sands J.M. (2006). Vasopressin increases plasma membrane accumulation of urea transporter UT-A1 in rat inner medullary collecting ducts. J. Am. Soc. Nephrol.

[11] Blount M.A., Mistry A.C., Fröhlich O., Price S.R., Chen G., Sands J.M., Klein J.D. (2008). Phosphorylation of UT-A1 urea transporter at serines 486 and 499 is important for vasopressin-regulated activity and membrane accumulation. Am. J. Physiol. Renal Physiol.

[12] Simons K., Gerl M.J. (2010). Revitalizing membrane rafts: New tools and insights. Nat. Rev. Mol. Cell Biol.

[13] Puri N., Roche P.A. (2006). Ternary SNARE complexes are enriched in lipid rafts during mast cell exocytosis. Traffic.

[14] Feng X., Huang H., Yang Y., Fröhlich O., Klein J.D., Sands J.M., Chen G. (2009). Caveolin-1 directly interacts with UT-A1 urea transporter: The role of caveolae/lipid rafts in UT-A1 regulation at the cell membrane. Am. J. Physiol. Renal Physiol.

[15] Chen G., Howe A.G., Xu G., Fröhlich O., Klein J.D., Sands J.M. (2011). Mature *N*-linked glycans facilitate UT-A1 urea transporter lipid raft compartmentalization. FASEB J.

[16] Huang H., Feng X., Zhuang J., Fröhlich O., Janet D., Klein J.D., Cai H., Sands J.M., Chen G. (2010). Internalization of UT-A1 urea transporter is dynamin dependent and mediated by both caveolae and clathrin coated pit pathways. Am. J. Physiol. Renal Physiol.

[17] Xu G., Su H., Carter B.C., Fröhlich O., Chen G. (2012). Depolymerization of cortical actin inhibits UT-A1 urea transporter endocytosis but promotes forskolin stimulated membrane trafficking. Am. J. Physiol. Cell Physiol.

[18] Hanzal-Bayer M.F., Hancock J.F. (2007). Lipid rafts and membrane traffic. FEBS Lett.

[19] Simons K., Toomre D. (2000). Lipid rafts and signal transduction. Nat. Rev. Mol. Cell Biol.

[20] Xiong Y., Antalffy G., Enyedi A., Strehler E.E. (2009). Apical localization of PMCA2w/b is lipid raft-dependent. Biochem. Biophys. Res. Commun.

[21] Hoover R.S., Poch E., Monroy A., Vázquez N., Nishio T., Gamba G., Hebert S.C. (2003). *N*-Glycosylation at two sites critically alters thiazide binding and activity of the rat thiazide-sensitive Na^+^:Cl^−^ cotransporter. J. Am. Soc. Nephrol.

[22] Lee T.K., Koh A.S., Cui Z., Pierce R.H., Ballatori N. (2003). *N*-glycosylation controls functional activity of Oatp1, an organic anion transporter. Am. J. Physiol. Gastrointest. Liver Physiol.

[23] Hendriks G., Koudijs M., van Balkom B.W., Oorschot V., Klumperman J., Deen P.M., van der Sluijs P. (2004). Glycosylation is important for cell surface expression of the water channel aquaporin-2 but is not essential for tetramerization in the endoplasmic reticulum. J. Biol. Chem.

[24] Vagin O., Kraut J.A., Sachs G. (2009). Role of *N*-glycosylation in trafficking of apical membrane proteins in epithelia. Am. J. Physiol. Renal Physiol.

[25] Scheiffele P., Peränen J., Simons K. (1995). *N*-glycans as apical sorting signals in epithelial cells. Nature.

[26] Chen G., Froehlich O., Yang Y., Klein J.D., Sands J.M. (2006). Loss of *N*-linked glycosylation reduces urea transporter UT-A1 response to vasopressin. J. Biol. Chem.

[27] Bradford A.D., Terris J.M., Ecelbarger C.A., Klein J.D., Sands J.M., Chou C.L., Knepper M.A. (2001). 97- and 117-kDa forms of collecting duct urea transporter UT-A1 are due to different states of glycosylation. Am. J. Physiol. Renal Physiol.

[28] Pech V., Klein J.D., Kozlowski S.D., Wall S.M., Sands J.M. (2005). Vasopressin increases urea permeability in the initial IMCD from diabetic rats. Am. J. Physiol. Renal Physiol.

[29] Parton R.G., Simons K. (2007). The multiple faces of caveolae. Nat. Rev. Mol. Cell Biol.

[30] Razani B., Lisanti M.P. (2001). Caveolins and caveolae: Molecular and functional relationships. Exp. Cell Res.

[31] Lajoie P., Nabi I.R. (2007). Regulation of raft-dependent endocytosis. J. Cell Mol. Med.

[32] Liu P., Rudick M., Anderson R.G. (2002). Multiple functions of caveolin-1. J. Biol. Chem.

[33] Lang T. (2007). SNARE proteins and ‘membrane rafts’. J. Physiol.

[34] Mistry A.C., Mallick R., Fröhlich O., Klein J.D., Rehm A., Chen G., Sands J.M. (2007). The UT-A1 urea transporter interacts with snapin, a SNARE-associated protein. J. Biol. Chem.

[35] Chamberlain L.H., Burgoyne R.D., Gould G.W. (2001). SNARE proteins are highly enriched in lipid rafts in PC12 cells: Implications for the spatial control of exocytosis. Proc. Natl. Acad. Sci. USA.

[36] Koticha D.K., Huddleston S.J., Witkin J.W., Baldini G. (1999). Role of the cysteine-rich domain of the t-SNARE component, SYNDET, in membrane binding and subcellular localization. J. Biol. Chem.

[37] Apodaca G. (2001). Endocytic traffic in polarized epithelial cells: Role of the actin and microtubule cytoskeleton. Traffic.

[38] Noda Y., Horikawa S., Katayama Y., Sasaki S. (2004). Water channel aquaporin-2 directly binds to actin. Biochem. Biophys. Res. Commun.

[39] Mazzochi C., Bubien J.K., Smith P.R., Benos D.J. (2006). The carboxyl terminus of the alpha-subunit of the amiloride-sensitive epithelial sodium channel binds to F-actin. J. Biol. Chem.

[40] Prat A.G., Cunningham C.C., Jackson G.R., Borkan S.C., Wang Y., Ausiello D.A., Cantiello H.F. (1999). Actin filament organization is required for proper cAMP-dependent activation of CFTR. Am. J. Physiol. Cell Physiol.

[41] Liedtke C.M., Hubbard M., Wang X. (2003). Stability of actin cytoskeleton and PKC-delta binding to actin regulate NKCC1 function in airway epithelial cells. Am. J. Physiol. Cell Physiol.

[42] Sadeghi A., Doyle A.D., Johnson B.D. (2002). Regulation of the cardiac l-type Ca^2+^ channel by the actin-binding proteins α-actinin and dystrophin. Am. J. Physiol. Cell Physiol.

[43] Kanzaki M., Pessin J.E. (2001). Insulin-stimulated GLUT4 translocation in adipocytes is dependent upon cortical actin remodeling. J. Biol. Chem.

[44] Simon H., Gao Y., Franki N., Hays R.M. (1993). Vasopressin depolymerizes apical F-actin in rat inner medullary collecting duct. Am. J. Physiol. Cell Physiol.

[45] Su H., Carter C.B., Fröhlich O., Cummings R.D., Chen G. (2012). Glycoforms of UT-A3 urea transporter with poly-*N*-acetyllactosamine glycosylation have enhanced transport activity. Am. J. Physiol. Renal Physiol.

[46] Blount M.A., Klein J.D., Martin C.F., Tchapyjnikov D., Sands J.M. (2007). Forskolin stimulates phosphorylation and membrane accumulation of UT-A3. Am. J. Physiol. Renal Physiol.

